# Towards a microRNA‐based Johne's disease diagnostic predictive system: Preliminary results

**DOI:** 10.1002/vetr.4798

**Published:** 2024-11-19

**Authors:** Paul Capewell, Arianne Lowe, Spiridoula Athanasiadou, David Wilson, Eve Hanks, Robert Coultous, Michael Hutchings, Javier Palarea‐Albaladejo

**Affiliations:** ^1^ School of Molecular Biosciences, College of Medical, Veterinary & Life Sciences University of Glasgow Glasgow UK; ^2^ Scotland's Rural College (SRUC) Edinburgh UK; ^3^ MI:RNA Edinburgh UK; ^4^ Department of Computer Science, Applied Mathematics and Statistics University of Girona Girona Spain

**Keywords:** Johne's disease, diagnostics, microRNA, predictive modelling

## Abstract

**Background:**

Johne's disease, caused by *Mycobacterium avium* subspecies *paratuberculosis* (MAP), is a chronic enteritis that adversely affects welfare and productivity in cattle. Screening and subsequent removal of affected animals is a common approach for disease management, but efforts are hindered by low diagnostic sensitivity. Expression levels of small non‐coding RNA molecules involved in gene regulation (microRNAs), which may be altered during mycobacterial infection, may present an alternative diagnostic method.

**Methods:**

The expression levels of 24 microRNAs affected by mycobacterial infection were measured in sera from MAP‐positive (*n* = 66) and MAP‐negative cattle (*n* = 65). They were then used within a machine learning approach to build an optimal classifier for MAP diagnosis.

**Results:**

The method provided 72% accuracy, 73% sensitivity and 71% specificity on average, with an area under the curve of 78%.

**Limitations:**

Although control samples were collected from farms nominally MAP‐free, the low sensitivity of current diagnostics means some animals may have been misclassified.

**Conclusion:**

MicroRNA profiling combined with advanced predictive modelling enables rapid and accurate diagnosis of Johne's disease in cattle.

## INTRODUCTION

Johne's disease is a chronic enteritis of ruminants caused by *Mycobacterium* avium subspecies *paratuberculosis* (MAP). It is of significant concern to the dairy industry as it can lead to decreased production, weight loss and death in infected cattle.^1^ Estimates of losses range from £10m in the UK^2^ to $200 m in the United States.^3^ Prevention and control of Johne's disease is difficult as MAP is environmentally resilient and vaccine efficacy is variable.^4^ As such, management and control strategies focus on testing and targeted removal of infected animals.^5^ Despite detection being a central component of control, diagnosis can be challenging as clinical signs are insidious and non‐specific.^1^ The most accurate diagnostic method to date is bacterial culture, but this method is costly and can take months to yield results.^6^ The most commonly used tests are serum antibody ELISAs, which have high specificity but low sensitivity in cattle (≈0.44), especially in the early stages of infection, leading to low accuracy (≈0.55).^6^, ^7^ Furthermore, it has been shown that MAP shedding may be occurring for up to 2 years before a positive milk ELISA result is obtained, resulting in high transmission rates that go undetected.^8^ Research is therefore needed to develop effective, early‐stage diagnostics and sustainable strategies to mitigate the economic impact of Johne's disease.

A promising approach for developing novel and reliable diagnostics is to harness the information contained in the expression profiles of small non‐coding RNA molecules. MicroRNAs (miRNAs) are found in most eukaryotes and play critical roles in gene regulation.^9^ They have diagnostic and prognostic value and are particularly suitable as biomarkers due to being stable and easily detectable in biofluids.^10^ Importantly, there have been several studies showing that expression levels of numerous miRNAs are altered by mycobacterial infection, including early‐stage^11^ and pre‐seroconversion MAP.^12^ Diagnostic accuracy, or the capacity of a test to discriminate diseased and healthy samples, can be further enhanced through the deployment of advanced statistical and machine learning (ML) methods within an artificial intelligence framework. These tools can significantly improve diagnosis by rapidly analysing experimental data, identifying patterns that could be missed by humans or less sophisticated methods, and producing predictions to support an objective assessment of the uncertainty regarding infection status. They have already proven useful for improving diagnosis and prognosis in human medicine, for instance in oncology.^13^

To explore the potential of miRNA profiling in Johne's disease diagnosis, this preliminary study measured the expression of selected miRNAs affected by mycobacterial infection in samples from MAP‐infected and uninfected cattle. These were processed through an ML pipeline for predictive modelling with the primary objective of building an optimal classifier for the diagnosis of Johne's disease using miRNA profiles.

## MATERIALS AND METHODS

### Sample collection

All cases submitted to the UK‐wide Premium Cattle Health Scheme between November 2021 and November 2022 with reported signs of Johne's disease were considered for inclusion in the study. MAP positivity was defined as animals with an ID Screen Paratuberculosis Indirect ELISA (Innovative Diagnostics) test result of greater than 70% (sample optical density divided by the positive control optical density, multiplied by 100) that either had a positive faecal PCR test within the subsequent 2 months or were from herds with over 3% MAP‐seropositive animals that had maintained this high level of herd seropositivity for at least the previous year. In total, 66 MAP‐positive cattle were identified over the sampling period. Sixty‐five MAP‐negative samples submitted to the same scheme were randomly selected as controls. To minimise false negatives, seronegative control samples were from herds that had no seropositive results in annual tests of all animals over 24 months of age for a minimum of five consecutive years.

### MicroRNA panel

miRNAs to be included in the MAP‐specific miRNA panel were selected through a review of 17 manuscripts identified in a PubMed‐based search using the terms ‘(((Johne's disease[Title/Abstract]) OR (Mycobacterium avium[Title/Abstract])) AND ((miRNA[Title/Abstract]) OR (microRNA[Title/Abstract])))’. Manuscripts were excluded if they were reviews, used a non‐bovine model, did not use blood or serum samples, were testing responses to interventions or were not directly assaying miRNAs. Candidate miRNAs were excluded if they were not present in the bovine miRNA repertoire reported by MIRBase^14^ or if there were significant differences between the probe sequences used and the target bovine miRNA in the database. These sequences were used to design a custom panel for the Fireplex miRNA platform (Abcam). Five miRNAs previously validated as standards in cattle were also included to act as normalisers for the expression data (Table 1). Signalling pathways targeted by each miRNA and the total panel were predicted using mirPathv3 (*p* < 0.05 threshold).^15^

**TABLE 1:** Summary information for the profiling panel, indicating the mature sequence and predicted signalling pathways targeted by each microRNA (miRNA) (*p* < 0.05). Normalizer miRNAs are indicated with an asterisk.

miRNA name	Mature sequence	Signalling pathways	Reference
bta‐miR‐105‐5p	UCAAAUGCUCAGACUCCUGUGGU	ErbB, mTOR	^16^
bta‐miR‐100‐5p	AACCCGUAGAUCCGAACUUGUG	Wnt, mTOR, Rap1	^17^
bta‐miR‐1247‐5p	ACCCGUCCCGUGCGUCCCCGGA	*	^16^
bta‐miR‐155‐5p	UUAAUGCUAAUCGUGAUAGGGGU	T cell receptor, B cell receptor, TGF‐beta	^18^
bta‐miR‐6517‐5p	UCAGGGUCCGUGAGCUCCUCGGC	*	^17^
bta‐miR‐24‐1‐5p	GUGCCUACUGAGCUGAUAUCAGU	ErbB	^17^
bta‐miR‐184‐3p	UGGACGGAGAACUGAUAAGGGU	*	^16^
bta‐miR‐142‐3p	AGUGUUUCCUACUUUAUGGAUG	*	^18^
bta‐miR‐137‐3p	UUAUUGCUUAAGAAUACGCGUAG	ErbB, MAPK	^16^
bta‐miR‐29b‐3p	UAGCACCAUUUGAAAUCAGUGUU	PI3K‐Akt, Thyroid hormone	^16^
bta‐miR‐582‐5p	UUACAGUUGUUCAACCAGUUACU	Wnt, Hippo, cGMP‐PKG	^11^
bta‐miR‐196b‐5p	UAGGUAGUUUCCUGUUGUUGGGA	ErbB	^16^
bta‐miR‐19b‐3p	UGUGCAAAUCCAUGCAAAACUGA	mTOR, Rap1, cAMP, GnRH	^17^
bta‐miR‐21‐5p	UAGCUUAUCAGACUGAUGUUGACU	Hippo, Jak‐STAT	^19^
bta‐miR‐133b‐3p	UUUGGUCCCCUUCAACCAGCUA	Adrenergic	^16^
bta‐miR‐378a‐3p	ACUGGACUUGGAGUCAGAAGU	*	^17^, ^18^
bta‐miR‐32‐5p	UAUUGCACAUGACUAAGUUGCAU	*	^17^, ^18^
bta‐miR‐202‐5p	UUCCUAUGCAUAUACUUCUUU	Prolactin, FoxO, Oestrogen	^16^
bta‐miR‐1271‐5p	CUUGGCACCUAGUAAGUACUCA	Oestrogen, Oxytocin	^17^
bta‐miR‐7857‐5p	AUAGCCAGUUGGGGAAGAAUGC	*	^17^
bta‐miR‐29a‐3p	CUAGCACCAUCUGAAAUCGGUUA	PI3K‐Akt, Thyroid hormone	^18^
bta‐miR‐433‐3p	AUCAUGAUGGGCUCCUCGGUGU	*	^16^
bta‐miR‐146a‐5p	UGAGAACUGAAUUCCAUAGGUUGU	*	^18^
bta‐miR‐301a‐3p	CAGUGCAAUAGUAUUGUCAAAGCAU	FoxO, p53, mTOR	^17^
*bta‐miR‐20a‐5p	UAAAGUGCUUAUAGUGCAGGUAG	*	^20^
*bta‐mir16a‐5p	UAGCAGCACGUAAAUAUUGGUG	*	^21^
*bta‐mir‐92a‐3p	UAUUGCACUUGUCCCGGCCUGU	*	^22^
*bta‐mir‐17‐5p	CAAAGUGCUUACAGUGCAGGUAGU	*	^23^
*bta‐mir‐93‐5p	CAAAGUGCUGUUCGUGCAGGUA	*	^24^

Abbreviations: cAMP, Cyclic Adenosine Monophosphate; ErbB, Erythroblastosis Oncogene B; GnRH, Gonadotropin‐Releasing Hormone; Hippo, Hippo; Jak‐STAT, Janus Kinase and Signal Transducer and Activator of Transcription; mTOR, Mammalian Target of Rapamycin; MAPK, Mitogen‐Activated Protein Kinase; Rap1, Ras‐association Proximate 1; PI3K‐Akt, Phosphoinositide 3‐Kinase and Protein Kinase B; Wnt, Wingless‐related Integration Site.

* Indicates no significant signalling pathway was reported by DIANA search.

### Expression analysis

To generate Fireplex miRNA profiles, 50 µL aliquots of serum from each sample were processed following the manufacturer's instructions, with hybridisation, melt‐off and capture temperatures of 39°C, 62°C and 39°C, respectively. Mean fluorescence intensities (MFIs) of miRNA‐specific particles per sample were measured using a Novocyte flow cytometer (Agilent). Raw FCS files were exported to Fireplex Analysis Workbench 2.0.274 (Abcam), and relative expression values were prepared using the geNorm function with pre‐selected normalisers.

### Supervised machine learning

Using the Fireplex‐processed and normalised (centred, scaled, Yeo‐Johnson's transformed) miRNA profiles as predictors and the established Johne's disease infectivity status as the response variable (infected vs. healthy sample), a preliminary investigation and benchmarking of plausible supervised ML methods for our predictive diagnosis aim was conducted. In line with previous reports showing its high predictive accuracy and applicability for medical diagnosis,^25^, ^26^, ^27^ the random forest (RF) algorithm stood out among the best performers. The RF method generated a diverse collection of decision trees trained on random subsets (resamples) of samples and miRNAs signals, with the predicted status for a sample being determined by majority vote across all trees. In this process, a score of variable importance in prediction (VIP) for the miRNA signals was determined by averaging how much the predictive accuracy changed after adding or removing each of them.

The training of the RF, including parameter tuning, was conducted through a five‐time repeated 10‐fold cross‐validation (CV) pipeline; that is, the input data were randomly partitioned into 10 folds, with nine folds used to train the model and one fold held out as the test set sequentially. Fold randomisation was repeated five times to reduce dependence on the initial partition. This helped to balance the variance bias trade‐off and to manage overfitting (where a method is overly specific to the training data); hence, contributing to a fairer assessment of their predictive ability on new samples. Performance metrics included those routinely used in classification tasks, including overall accuracy, area under the receiver operating curve (AUC), sensitivity and specificity. All measures ranged from 0 to 1, with values closer to 1 indicating better performance (95% confidence intervals given in parenthesis). These were assessed on each test set across the CV process and then averaged to provide an overall assessment of performance on independent blind samples. Our RF training was based 500 decision trees grown in each CV round, with AUC used to tune by random search the number of random predictors included at each split in a tree (*mtry* tuning parameter, optimal value = 13). Predicted status probabilities for the given samples were obtained from the trained RF by averaging across CV rounds. This analysis was implemented using our own computing pipelines written using the R system for statistical computing v4.2.1,^28^ with the ML methods implemented using the package Caret v6.0‐94 and performance measurements based on the package MLeval v0.3. Extensive details about the ML approach and assessment of classification algorithms can be found elsewhere (see refs.^29^, ^30^, ^31^).

## RESULTS

**FIGURE 1:**
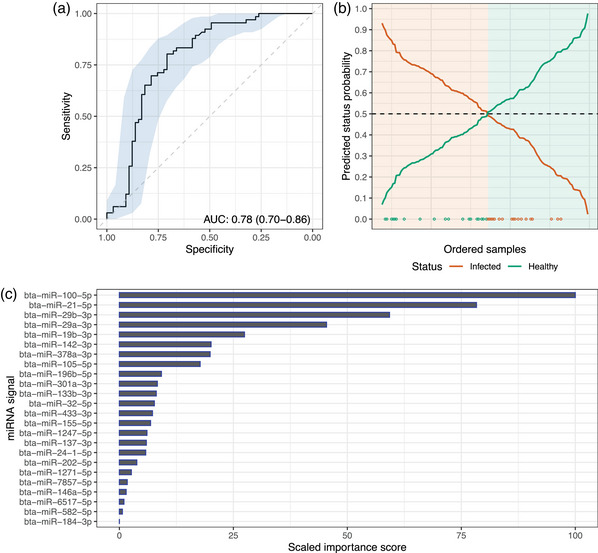
Random forest classifier results: (a) receiver operating curve and area under the curve (AUC). (b) Probabilities of status (infected/healthy) for collected cattle samples (samples ordered from left to right according to probability, status allocation based on the usual 0.5 probability threshold, background colour indicates allocated infectivity status, points at the bottom indicate misclassified samples with colour corresponding to actual status). (c) Ranking of miRNA signals according to importance in discriminating infectivity status

A review of the literature, including experiments conducted with cattle and MAP, suggested 24 miRNAs with altered expression in response to mycobacterial infection (Table 1).^16^, ^17^, ^32^ Across the total panel, there was enrichment for miRNA‐target genes involved in PI3KT, oestrogen, ErbB and FoxO signalling (*p* < 0.05). After the MFI of each miRNA was measured across all samples, the mean coefficient of variation (CoV) was found to be 0.49 (0.38‒60) for normalising miRNAs and 1.23 (0.82‒1.64) for diagnostically informative miRNAs. The lower CoV indicated that the miRNAs selected for normalisation were stable and suitable standards. The RF trained on pre‐processed miRNA profiles provided cross‐validated accuracy of 0.72 (0.70‒0.74), with this being statistically significantly greater than the naïve classifier accuracy (0.50; *p* < 0.001). Using infected status as the reference class, an AUC of 0.78 (0.70‒0.86) was reached (Figure 1a), with a sensitivity of 0.73 (0.61‒0.82) and a specificity of 0.71 (0.59‒0.80). Misclassification was more common for healthy samples (Figure 1b; 14.96% healthy classed as MAP‐infected against 12.67% infected classed as healthy). The ranking of the most relevant miRNAs to discriminate infection status was led by (RF VIP scores scaled into [0, 100] shown in parenthesis): bta‐miR‐100‐5p (100), bta‐miR‐21‐5p (78.32), bta‐miR‐29b‐3p (59.25) and bta‐miR‐29a‐3p (45.44). Moreover, the least contributing ones were: bta‐miR‐184‐3p (0.00), bta‐miR‐582‐5p (0.66), bta‐miR‐6517‐5p (0.99) and bta‐miR‐146a‐5p (1.47) (Figure 1c).

## DISCUSSION

The aim of this study was to evaluate the potential for a selected miRNA expression panel to be used in combination with modern ML technologies to build a diagnostic testing tool for discrimination between MAP‐positive and MAP‐negative bovine sera.

Our preliminary results indicate that a panel of 24 miRNAs, previously identified as having altered expression in MAP infection, processed by a tailored RF algorithm (one of the most popular ML methods) was able to achieve an accuracy of 0.72 (sensitivity 0.73; specificity 0.71) in discriminating between healthy and MAP‐infected cattle. It is possible that specificity is underestimated in our dataset since, despite control animals being screened with a high specificity (>0.998),^33^ ELISA sensitivity is regarded low, especially in the early stages of infection.^6^, ^7^ Efforts were made to minimise this issue by only including control cattle from low‐risk farms, but some misclassification may have occurred due to the presence of undiagnosed MAP‐positive animals within these herds, given the low sensitivity of current ELISA screening methods. In future trials, cattle can be diagnosed using more accurate culturing or PCR techniques to overcome this shortcoming. Another possible issue may be diagnostic cross‐reactivity with other mycobacterial species, such as *Mycobacterium tuberculosis* (TB). Cross‐reactivity can be negated by including MAP‐ or gut‐infection‐specific miRNAs, but future studies will include challenging the model with known TB‐positive sera. We hypothesise that comparing the miRNA profiles of MAP, TB and co‐infected cattle will allow for the training of models able to discern species, as well as infection status.

In the current analysis of the 24 miRNAs identified in previous Johne's disease studies, bta‐miR‐100‐5p and bta‐miR‐21‐5p emerged as the most prominent biomarkers, whereas others, such as bta‐miR‐582‐5p and bta‐miR‐184‐3p, showed a less significant contribution. Although these miRNAs have been associated with Johne's disease in prior research and likely play a role in the complex biological processes of the disease, our findings suggest that some offer better diagnostic potential than others. However, these results are based on a limited sample size, which underscores the need for further validation and extended trials to fully elucidate their potential as biomarkers. In addition to widening the scope of the assay, accuracy may be improved by including additional informative markers. The current panel was suggested by a limited literature review and included target pathways known to be impacted by Johne's disease, including PI3KT, oestrogen, ErbB and FoxO signalling.^34^, ^35^ The panel could be expanded by including data from additional mycobacterial species, examining miRNAs known to target additional MAP‐affected pathways or performing a hypothesis‐generating study using miRNASeq. miRNAs may also improve the MAP diagnosis pipeline by exploiting their stability in biofluids, particularly milk.^3^, ^36^ Most commercial tests for Johne's disease use sera, adding expertise and cost barriers to screening. The feasibility of profiling milk miRNAs for MAP diagnosis is currently being evaluated. Finally, although primarily an infection of cattle, MAP also affects other ruminants.^1^ miRNAs are highly conserved between species, suggesting that the current miRNA panel would be suitable for sheep and goats with small adjustment, although species‐specific trained models would be required. The capacity to provide a single diagnostic that allows for pathogen detection and identification across multiple species using an easily accessible biofluid makes miRNA profiling a highly attractive tool for veterinary diagnosis.

In summary, this study indicates that miRNA profiling combined with advanced predictive modelling has the potential to serve as a diagnostic test for Johne's disease in cattle. Efforts are currently ongoing to expand and validate the method with more sample data, which will help to further improve precision and expand the approach to characterise Johne's disease stage and enhance early‐stage diagnosis, including the possibility of identifying MAP‐positive animals prior to seroconversion, and distinguish between alternative mycobacterial pathogens through miRNA diagnostics methods. This technology is patent pending.

## AUTHOR CONTRIBUTIONS

*Conceptualisation and funding acquisition*: Eve Hanks, Javier Palarea‐Albaladejo and Robert Coultous. *Methodology*: Javier Palarea‐Albaladejo, Paul Capewell and Robert Coultous. *Formal analysis and software*: Javier Palarea‐Albaladejo and Paul Capewell. *Investigation and data curation*: Arianne Lowe, David Wilson, Michael Hutchings and Spiridoula Athanasiadou. *Supervision*: Eve Hanks, Javier Palarea‐Albaladejo, Michael Hutchings and Robert Coultous. *Writing*: all authors.

## CONFLICT OF INTEREST STATEMENT

E. Hanks and R. Coultous are employees of MI:RNA, and P. Capewell and J. Palarea‐Albaladejo have acted as paid consultants for MI:RNA. The remaining authors declare that they have no commercial or financial relationships that could be construed as a potential conflict of interest.

## ETHICS STATEMENT

The use of historic sera was considered by the SRUC Ethical Committee, and permission was granted as samples had been submitted with farmers' consent via the Premium Cattle Health Scheme.

## Data Availability

The data that support the findings of this study can be made available with the permission of MI:RNA. Reasonable requests should be addressed to robert.coultous@mirna-diagnostics.com.
